# An exploration of the impact of contextual information on the emotion recognition ability of autistic adults

**DOI:** 10.1002/ijop.12834

**Published:** 2022-02-14

**Authors:** Dale Metcalfe, Karen McKenzie, Kristofor McCarty, Thomas V. Pollet, George Murray

**Affiliations:** ^1^ Northumbria University Newcastle upon Tyne UK; ^2^ NHS Lothian Edinburgh UK

**Keywords:** Autism spectrum disorder, Emotion recognition, Context

## Abstract

Studies of non‐autistic individuals and people with an intellectual disability show that contextual information impacts positively on emotion recognition ability, however, this area is not well researched with autistic adults. We investigated this using a static emotion recognition task. Participants completed an emotion recognition task in person or online. In total, 46 autistic participants and 379 non‐autistic participants completed the task. A linear mixed model showed that autistic adults had significantly lower accuracy when identifying emotions across all contexts, compared to control participants, even when contextual information was present. No significant effect of context was found in either group, nor was gender shown to be an influential variable. A supplementary analysis showed that higher scores on the Autism‐Spectrum Quotient led to lower scores on the emotion recognition task; no effect of context was found here either. This research adds to the limited work investigating the influence of contextual factors in emotion recognition in autistic adults. Overall, it shows that context may not aid emotion recognition in this group in the same way as it does for non‐autistic individuals.

Recognising emotional expression is important for human communication and social interaction (Harms et al., 2010). Research investigating people's recognition of emotions shows various factors influence the interpretation of emotions. One such factor is autism spectrum disorder (ASD), a condition recognised and diagnosed based on persistent deficits in social communication and interaction, as well as demonstrating restricted, repetitive patterns of behaviour (American Psychiatric Association, 2013). Henceforth ASD is referred to as autism, and “autistic people” are referred to in identity‐first language.

Several reviews and meta‐analyses show that overall, using a variety of different tasks, autistic people show reduced accuracy when recognising emotions (Harms et al., 2010; Uljarevic & Hamilton, 2013). A further review (Black et al., 2017) concluded that the cognitive processes employed by autistic people are atypical, but patterns across studies are inconsistent. These results extend to the non‐autistic population, with autistic traits predicting emotion recognition ability; people with higher autistic traits perform more poorly overall (e.g., Martin et al., 2019). Other studies, however, show that autistic adults (e.g., Adolphs et al., 2001) and children (e.g., Shanok et al., 2019) perform comparably to non‐autistic peers.

These differences in emotion recognition have been attributed to various factors. One argument is that autistic people attend the eye regions of stimuli differently, which has been evidenced across several studies (e.g., Kliemann et al., 2012). Cuve et al.'s (2018) systematic review shows that autistic adults typically attend the eye region differently to non‐autistic people; most studies indicate reduced attenuation of the eyes overall and initially. These factors potentially lead to information loss and reduce the accuracy of emotion recognition. Other potential mechanisms include atypical brain activity, evidenced by electroencephalography studies (Black et al., 2017). However, caution should be exercised here due to the heterogeneity of autistic individuals and that these regions may be responsible for other processes; other tasks requiring these regions are unaffected (South et al., 2008).

Disparity in findings may be attributable to methodological differences between studies, particularly the nature of the tasks used. Some studies ask participants to match pre‐existing labels to emotion stimuli, while others require participants to create their own labels. The former approach may allow participants to employ compensatory mechanisms (Harms et al., 2010), thus no difference may be found between autistic and non‐autistic individuals in some cases. Other evidence has shown that the type of stimuli used has a role to play. Photographs lead to poorer emotion recognition in autistic children while presenting cartoon‐like images reveal no difference (Rosset et al., 2008). Differences exist between static and dynamic stimuli too, with autistic people being disadvantaged when dynamic stimuli are used (Enticott et al., 2014). Individual differences, such as age and gender, also appear to influence emotion recognition. For both autistic and non‐autistic people, emotion recognition abilities improve throughout childhood and adolescence (Thomas et al., 2007) and women perform better than their male peers on emotion recognition tasks (Sucksmith et al., 2013).

Further individual differences to consider are intelligence, familiarity, anxiety and co‐occurring alexithymia. Intelligence has been shown to predict emotion recognition (for review see Schlegel et al., 2020), however, a meta‐analysis indicates that it does not account for emotion recognition impairments experienced by autistic people (Lozier et al., 2014). Familiarity is a known influence on emotion recognition, particularly for autistic people. There is evidence that autistic and non‐autistic people perform comparably when viewing stimuli featuring familiar people, such as their parents (Shanok et al., 2019). However, when the people in the stimuli are unfamiliar, group differences are revealed in which autistic people identify the emotions less accurately than their non‐autistic peers (Harms et al., 2010; Pierce & Redcay, 2008; Shanok et al., 2019). This implies that the people featured in the stimuli are important when considering group differences. People with anxiety are more sensitive to negative emotions (e.g., Gutiérrez‐García & Calvo, 2017), however, this can lead to misclassification of other observed emotions (Heuer et al., 2010). Autistic individuals may also be affected, (e.g., avoiding viewing faces to avoid anxiety Harms et al., 2010) but specific research is lacking. One notable study found that, in children, anxiety was unrelated to emotion recognition ability (Wong et al., 2012). Lastly, alexithymia is an inability to identify and describe one's own emotions (Bird & Cook, 2013) and is experienced by many autistic people (Kinnaird et al., 2019), while remaining distinct from autism itself (Cuve et al., 2021). Crucially, alexithymia has been shown to predict a person's emotion recognition ability, with those with the condition performing worse on emotion recognition tasks (Ola & Gullon‐Scott, 2020).

Despite some inconsistencies in s from existing studies, overall, it appears that emotion recognition may be challenging for some autistic people. As such, a range of interventions have been developed. Reviews of interventions for both emotion recognition (Berggren et al., 2018; Kouo & Egel, 2016) and social skills (Jonsson et al., 2016) show that, while there are benefits, the long‐term impact and generalisability of these programmes are not established. Kouo and Egel (2016) noted that one factor which may have impacted the effectiveness of emotion recognition interventions was the type and amount of contextual information available within the learning process.

A review by Barrett et al. (2011) highlighted that contextual information can be communicated in different ways including body language, situational cues, movement and sound within stimuli. This concludes that context is highly influential, that its integration into emotion processing happens automatically and early in the process. Research with atypical populations into the role of context in emotion recognition is limited and the s are less conclusive. Two studies including adult participants with an intellectual disability (*M* = 35 and *M* = 45, respectively), found contextual information can aid emotion recognition (McKenzie et al., 2001; Scotland et al., 2016), however, another with children (*M* = 12 years) found absence of contextual information led to better emotion recognition (Murray et al., 2018).

Early research with autistic children (*M* = 14 years) found that they matched facial expressions to contexts less accurately than their non‐autistic peers (Hobson, 1986). A later study found that the presence of context did not help young people with autism (7–16 years) to recognise emotions (Wright et al., 2008). A study of children and adolescents (*M* = 10 years), showed contextual cues aid emotion recognition for both autistic and non‐autistic people; however, this study used stimuli that did not depict facial expressions (Metcalfe et al., 2019). Sasson et al. (2016) found no significant differences in the emotion recognition of autistic adults (*M* = 23 years), compared to non‐autistic controls (*M* = 35 years), when faces were presented in isolation; but the former performed more poorly when emotions were presented in context. These s highlight a lack of clarity about context's role in emotion recognition for autistic people. Although it appears to be less helpful for autistic, compared to non‐autistic, adults.

Intervention studies suggest that context may be a relevant factor. Golan et al. (2010) conducted an intervention where children watched video clips of animated vehicles with human faces which depicted and drew attention to different emotions across situations. The research found children with autism who took part in the intervention increased their use of emotion‐relevant vocabulary post‐intervention. It was suggested that part of the reason why their intervention was successful in improving emotion recognition was the reduction in demands from contextual information. For example, the trains ran on tracks which restricted the range of situations within which emotions could occur. Furthermore, emotions were explicitly identified and explained in context, thereby reducing the need to interpret the contextual information.

Similarly, Conallen and Reed (2016), in an emotion recognition intervention for children with autism (*N* = 10), explicitly linked contextual information to emotions. Black and white cartoon‐based line drawings were used to teach the children emotional vocabulary. Following the intervention, participants were able to link the drawings depicting each emotion to an appropriate context where the emotion might be expressed. They could subsequently generalise to untrained pictures. While this study had a small sample size and the long‐term impact of the intervention is unclear, the s suggest that the type, amount and presentation of contextual information used may be important.

In summary, contextual information may play an important role within socio‐emotional interventions. At present, however, the explicit role of context in facilitating the emotion recognition of autistic people, particularly adults, is underexplored. Similarly, the interplay between autistic traits and context in the adult population remains unclear. As differences in emotion recognition occur throughout development (e.g., Thomas et al., 2007) and both gender and age appear to impact emotion recognition (e.g., Sucksmith et al., 2013), the current study will focus on adults and investigate the role of gender. While intelligence, anxiety and alexithymia all have a role to play, due to time constraints within testing sessions, these were not explored in the current study. Familiarly was also not investigated, the stimuli were unfamiliar to all participants.

This study aims to address the question: how do autistic adults perform on an emotion recognition task when the level of contextual cues is changed? Second, the study will examine the relationship between autistic traits and the role of context. It is hypothesised that:

H1. Non‐autistic people will more accurately identify the emotions than autistic people.

H2. Women will identify the emotions more accurately than men.

H3. Autistic people will show greater accuracy when contextual information is absent.

H4. People with lower autistic traits will identify emotions more accurately than people with higher autistic traits.

H5. People with higher autistic traits will show greater accuracy when contextual information is absent.

## METHOD

### Design

The study used a nested design, whereby participants completed multiple emotion recognition trials, therefore, trial responses are nested in participants. Performance on the emotion task was used as the outcome (dependent) variable and was predicted by the set of explanatory (independent) variables.

Ethical approval was granted from Northumbria University's Health and Life Sciences Ethics Committee. Informed consent was obtained from all participants in this study. Procedures performed in this research involving human participants were in line with the 1964 Helsinki Declaration and its later amendments or comparable ethical standards. All participants included in the study provided informed consent either in writing, or as part of the online questionnaire.

### Materials

First, participants provided demographic information (e.g., diagnosis, gender) which were used as explanatory variables.

**Figure 1:**
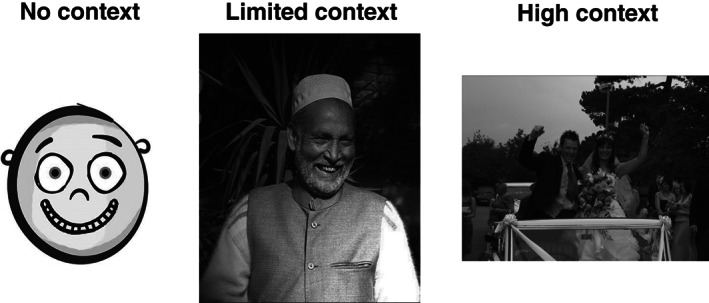
Examples of stimuli used at each level of context for the emotion “Happy.” *Source*: http://www.flickr.com/photos/kkoshy/2460058549; http://www.flickr.com/photos/rileyroxx/225440099.

Second, participants completed an emotion recognition task, used previously in other studies of emotion recognition (see McKenzie et al., 2020). This contains nine different emotions (happy, sad, neutral, surprise, disgust, bored, angry, scared and worried) and there are three stimuli for each emotion (one per level of context—see below); there are 27 trials in total. Labels for emotions were not revealed to participants. Participants were asked to name emotions in a free‐labelling format, whereby they were presented with a textbox to write their answer in while viewing the stimuli. These trials were presented in blocks, displaying either *no context*, *limited context* or *high context* stimuli. *No context* presented coloured line drawings of a face. *Limited context* presented colour photographs with limited information that was relevant to the emotion being displayed. *High context* presented colour photographs of emotions being displayed in situations that were congruous with the target emotion, for example, a couple looking happy at their wedding (Figure 1). Blocks were presented in a fixed order. Emotions within each were presented in a fixed sequence that differed between blocks. For further details, see https://osf.io/gm9eu/.

This task was scored in a semi‐automated way using a Python script that compared each word in the participant's answers against a list of acceptable (synonyms) words for each emotion. If a given answer was on this list their answer was marked as correct. If not, it was checked against all other emotion lists and marked incorrect if present (e.g., a happy emotion being labelled as sad), or on a list of words that were never correct (e.g., “unsure”). A response that was not in any of the aforementioned lists, was manually marked by the researchers as either correct or incorrect. These responses were then added to the relevant list. The initial lists of correct and incorrect words were constructed and agreed upon prior to the scoring process and then checked again at the end by multiple members of the research team.

Correct answers were scored as 1 and incorrect as 0. Performance on this task was the predictor variable. The level of contextual information was an explanatory variable.

Lastly, some participants were asked to complete the original Autism‐Spectrum Quotient (AQ; Baron‐Cohen et al., 2001). This is a measure of autistic‐like traits and is designed for people with average intelligence and reading ability. Each item is a statement about the person's personality, which they respond to on a four‐point Likert scale (definitely agree to definitely disagree). Scores are collapsed into either indicating (score = 1) or not indicating (score = 0) an autistic‐like trait, ing scores are summed. Possible scores range from 0 to 50, with higher scores indicating greater autistic‐like traits. Baron‐Cohen et al. (2001) found that 80% of autistic people scored 32+, while only 2% of the non‐autistic participants scored this high. In the current study, Cronbach's alpha showed good internal consistency (*α* = .87).

### Procedure

Participants were recruited either online (via social media) or in person. Prospective participants were provided with information about the study, a link to record consent and access to the study materials. Those who took part offline were provided a paper equivalent. Autistic participants were recruited online through social media and an autism support charity. Autism diagnosis was self‐reported and not independently confirmed by the researchers, however, the charity only provided support to people with a confirmed diagnosis of autism.

### Participants

A total of 546 participants started the survey and upon removal of incomplete data, 425 participants remained. Forty‐six indicated that they were autistic (male = 26, female = 16, other = 4; *M* = 28.78 years, *SD* = 9.83). Of these, 26 participants were recruited from a charity supporting autistic people (formal diagnosis required to attend); 20 indicated they were autistic when providing demographic information. Of the latter, on the AQ, 4 participants scored below the threshold of 32 (scoring 21, 26, 27, 29), 10 scored above 32, and 6 did not complete the AQ. The non‐autistic group comprised 379 participants (male = 91, female = 285, other = 3; *M* = 29.47 years, *SD* = 13.34). Eight autistic people reported an additional diagnosis: 6 “learning difficulty” and 2 “other.” Thirty‐three non‐autistic people reported additional diagnoses: 17 “learning difficulty,” 9 “physical disability” and 7 “other.” Participants were excluded if they had a severe visual impairment or a condition with a strong evidence base related to emotion recognition impairment other than autism (e.g., intellectual disability).

### Analysis

As the study was a nested design, with 27 trials nested in each of the 425 participants, a multilevel logistic model with a random intercept, at participant level, was applied. This approach is suggested to have greater statistical power compared to other traditional approaches, such as analysis of variances (Hoffman & Rovine, 2007).

The approach is to build a model that predicts the outcome variable using the set of explanatory variables. The explanatory (independent) variables were diagnosis (autistic or not), level of contextual information (no, limited and high) and gender. The outcome (dependent) variable was correct response at trial level (0 = incorrect, 1 = correct). As the outcome variable is dichotomous, a logistic model was used. First, a null model was created with no explanatory variables. Subsequently, explanatory variables were added incrementally based on the hypotheses. First adding diagnosis, followed by context, then gender and finally an interaction between diagnosis and context. This allows the construction of a comprehensive model that reveals which variables influence the outcome. A subset of data was analysed where AQ scores had been gathered from the participants. Here the same analyses were run but with AQ score in place of diagnosis. Analyses were run in R‐3.5.1 (with Ime4; Bates et al., 2012). For details, see https://osf.io/gm9eu/.

## RESULTS

### Autism diagnosis analysis

Table 1 contains means and standard deviations, and correlations between variables in the study. Correlations were run to explore relationships between these variables, whereby dichotomous variables were treated as continuous variables (Khamis, 2008). Context was not included as it contained three categories and was a within‐participant variable, which could therefore only correlate with the proportion of correctly identified emotions. Correlations indicated a relationship between diagnosis and gender, and diagnosis and correctly identified stimuli.

**TABLE 1:** Means, standard deviations and correlations with confidence intervals

Variable	M (SD)	Age	Gender	Autism diagnosis
Age	29.40 (13.00)			
Gender	1.74 (0.47)	−.01 [−.11, .08]		
Autism diagnosis	0.11 (0.31)	−.02 [−.11, .08]	−.16** [−.25, −.07]	
Proportion correct	0.67 (0.15)	−.05 [−.15, .04]	.10* [.00, .19]	−.22** [−.31, −.13]

*Note*: Coding for gender is: 1 = male, 2 = female. The coding for autism diagnosis is: 0 = non‐autistic, 1 = autistic. Proportion correct refers to the mean score of correctly identified emotions across trials: 0 = incorrect, 1 = correct. Values in square brackets indicate the 95% CI.

CI = confidence interval.

* *p* < .05.

** *p* < .01.

**Figure 2:**
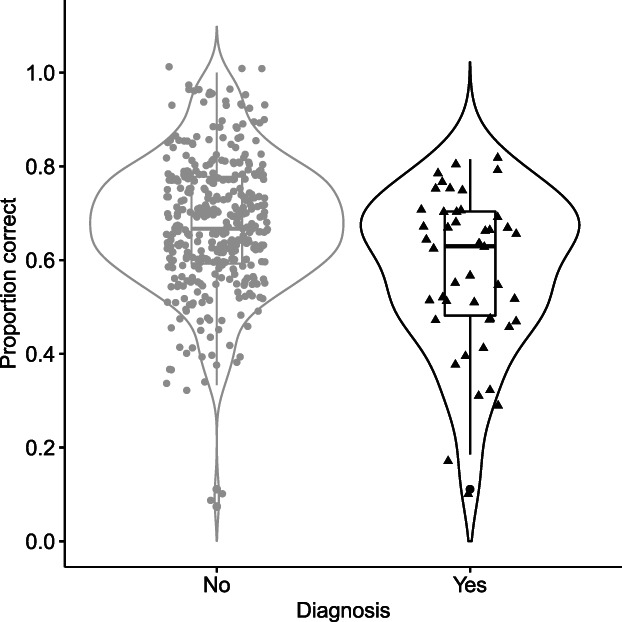
Violin plots showing the results of model 2.

Change in model fit was judged according to change in fit criteria (Burnham & Anderson, 2004). The null model was substantially improved upon with the inclusion of autism diagnosis (∆AIC = 18; ∆BIC = 11; ∆ ≈ 10 is suggested to be a very strong improvement). The addition of other variables did not improve model fit, meaning the inclusion of level of context does not improve modelling of participants correctly identifying emotions. Model 2 shows the best fit, and odds ratios indicate that autism diagnosis decreases the odds of correctly identifying emotions by 1.61 (1/0.620). In all, autism diagnosis is associated with poorer emotion recognition. Figure 2 summarises this finding (Tables 2 and 3).

**TABLE 2:** Model summaries of diagnosis multilevel logistic models (fixed effects)

	Correct				
	Model 1	Model 2	Model 3	Model 4	Model 5
Autism diagnosis		−0.478***	−0.478***	−0.454***	−0.376**
Context: limited			0.035	0.035	0.038
Context: high			0.074	0.074	0.107*
Gender				0.095	
Autism diagnosis × context: medium					−0.027
Autism diagnosis × context: high					−0.276
Intercept	0.756***	0.807***	0.770***	0.603***	0.759***
*N*	11,475	11,475	11,475	11,475	11,475
Log‐likelihood	−7139.344	−7129.147	−7128.051	−7127.141	−7126.120
AIC	14,282.690	14,264.300	14,266.100	14,266.280	14,266.240
BIC	14,297.390	14,286.340	14,302.840	14,310.370	14,317.680

*Note*: Components included in model can be identified by the presence of a complete cell in a particular row. Model 1 is the null model. Reference category for “context” is none.

AIC = Akaike's information criteria; BIC = Bayesian information criteria.

* *p* <.05.

** *p* <.01.

*** *p* <.001.

**TABLE 3:** Odds ratios for selected models in the diagnosis analysis, corresponding to Table 2

	Correct			
	Model 2	Model 3	Model 4	Model 5
Autism diagnosis	0.620***	0.620***	0.635***	0.686**
Context: limited		1.036	1.036	1.039
Context: high		1.077	1.077	1.113*
Gender			1.099	
Autism diagnosis × context: medium				0.973
Autism diagnosis × context: high				0.758

*Note*: Components included in model can be identified by the presence of a complete cell in a particular row. Model 1 is the null model. Reference category for “context” is none.

* *p* <.05.

** *p* <.01.

*** *p* <.001.

### AQ analysis

The results from a sub‐set of 194 participants who had provided complete AQ responses were analysed. Table 4 shows the means, standard deviations and correlations between variables in the study. Correlations indicated a relationship between correctly identified stimuli, and both diagnosis and AQ score. There was also a moderately strong correlation between AQ score and diagnosis, and for this reason we decided not to include both variables in a model, but instead focus on AQ in this analysis.

**TABLE 4:** Means, standard deviations and correlations with confidence intervals

Variable	M (SD)	Age	Gender	Autism diagnosis	AQ score
Age	29.54 (13.25)				
Gender	1.72 (0.51)	.04 [−.10, .18]			
Autism diagnosis	0.15 (0.36	.02 [−.12, .16]	−.13 [−.26, .01]		
Proportion correct	0.66 (0.13)	−.20** [−.33, .06]	.08 [−.06, .22]	−.21** [−.34, −.07]	
AQ score	18.79 (8.41)	−.00 [−.14, .14]	−.08 [−.22, .06]	.56** [.45, .65]	−.24** [−.37, −.10]

*Note*: Coding for gender is: 1 = male, 2 = female. The coding for autism diagnosis is: 0 = non‐autistic, 1 = autistic. Proportion correct refers to the mean score of correctly identified emotions across trials: 0 = incorrect, 1 = correct. Values in square brackets indicate the 95% CI.

AQ = Autism‐Spectrum Quotient; CI = confidence interval.

* *p* <.05.

** *p* <.01.

As before, change in model fit was judged according to change in fit criteria (AIC/BIC; Burnham & Anderson, 2004). The null model was substantially improved upon with the inclusion of AQ score (∆AIC = 10; ∆BIC = 13). Addition of other variables, including context, did not improve model fit, which indicates context was not associated with emotion recognition accuracy. Model 2 shows the best fit, and odds ratios indicate that 1 point increase on the AQ decreases the odds of correctly identifying emotions by a factor of 1.02 (1/0.983; i.e., a decrease in the odds of recognition by a factor of 1.02 per 1 AQ point increase). In all, higher AQ score diagnosis is associated with poorer emotion recognition (see Table 5 for logit coefficients and model fit; see Table 6 for an overview of odds ratios).

**TABLE 5:** Model summaries of AQ score multilevel logistic models (fixed effects)

	Correct				
	Model 1	Model 2	Model 3	Model 4	Model 5
AQ		−0.018***	−0.018***	−0.017***	−0.014
Context: medium			−0.019	−0.019	−0.045
Context: high			0.059	0.059	0.303
Gender				0.077	
AQ × context: medium					0.001
AQ × context: high					−0.013
Constant	0.717***	1.048***	1.035***	0.895***	0.964***
*N*	5238	5238	5238	5238	5238
Log‐likelihood	−3303.079	−3297.288	−3296.672	−3296.257	−3295.085
AIC	6610.158	6600.576	6603.344	6604.513	6604.170
BIC	6623.286	6620.267	6636.162	6643.895	6650.115

*Note:* Components included in model can be identified by the presence of a complete cell in a particular row. Model 1 is the null model. Reference category for “context” is none. ‘AIC’ indicates Akaike's Information Criteria; AQ = Autism‐Spectrum Quotient; ‘BIC’ indicates Bayesian Information Criteria.

* *p* <.05.

** *p* <.01.

*** *p* <.001.

**TABLE 6:** Odds ratios for selected models in the AQ score analysis, corresponding to Table 5

	Correct			
	Model 2	Model 3	Model 4	Model 5
AQ	0.983***	0.983***	0.983***	0.986
Context: medium		0.982	0.982	0.956
Context: high		1.061	1.061	1.354
Gender			1.080	
AQ × context: medium				1.001
AQ × context: high				0.987

*Note:* Components included in model can be identified by the presence of a complete cell in a particular row. Model 1 is the null model. Reference category for “context” is none.

AQ = Autism‐Spectrum Quotient.

* *p* <.05.

** *p* <.01.

*** *p* <.001.

## DISCUSSION

This study contributes to the small body of existing research investigating contextual information's role in the emotion recognition of autistic adults. The study was designed to reduce compensatory mechanisms through its use of a free labelling task (Harms et al., 2010) and, as it is influential (Sucksmith et al., 2013), the role of gender. The stimuli in the study were comparable to those in interventions and assessments of emotion recognition, used in clinical and educational settings (e.g., Murray et al., 2018).

Hypotheses H1 and H4, which states that non‐autistic people and people with lower autistic traits will more accurately recognise emotions than autistic people and people with higher autistic traits, were supported; findings which are consistent with past research (see Martin et al., 2019; Uljarevic & Hamilton, 2013). Decoding emotions is central to social interaction (Harms et al., 2010), therefore, the results suggest that autistic adults may be at a disadvantage in social relationships. They also indicate a greater number of autistic traits leads to poorer emotion recognition, however, this effect is across both autistic and non‐autistic participants. It is not possible to assess if this effect holds for autistic people specifically from the current research, due to the relatively low number of autistic participants with complete AQ responses. No support was found for the second hypothesis H2, which states that females will show superior emotion recognition skills, as no effect of gender was found. While gender may be influential in other studies, most of the variation in the current sample is attributed to the person's diagnostic status. It should be noted that compared to the previous research which has investigated context's role, the autistic adults in the current study are older than those in other studies (see Hobson, 1986; Metcalfe et al., 2019; Sasson et al., 2016; Wright et al., 2008).

The main aim of the study was to explore the role of contextual information on emotion recognition in autistic adults. Hypotheses H3 and H5, which states that autistic people and people with higher autistic traits will show greater accuracy in the absence of contextual information, was rejected as no effect of contextual information on emotion recognition ability was found. The present study adds to the limited research in this area, demonstrating contextual cues do not aid autistic adults identify emotions. Sasson et al. (2016) found that autistic people performed poorer than non‐autistic people in the presence of context and the current research makes the same finding. Unlike Sasson et al., the present study showed that emotion recognition by autistic people was also poorer in the absence of contextual cues. Furthermore, non‐autistic people recognised emotions more accurately, in line with previous research (see Uljarevic & Hamilton, 2013).

Despite past research indicating that contextual information influences the emotion recognition (Barrett et al., 2011), the present study found no such effect. This may be due to the task that was used. While previous research using the same task found emotion recognition varied according to context, this was primarily with children and people with an intellectual disability (McKenzie et al., 2001; Scotland et al., 2016), unlike the adults without learning disability in the current research. This suggests that the impact of context, and the most helpful level of context, may depend upon the person's ability to ignore extraneous cues irrelevant to the emotion (Murray et al., 2018). While stimuli revealed differences between autistic and non‐autistic participants, the present study suggests that other group‐level factors may also be relevant. The older age and higher ability of all participants may have meant that they were able to focus on relevant cues and ignore extraneous information, across all levels of context. This is, however, speculative as no measures of executive ability or IQ were used in this study. Crucially, the fact that context impacts results differently across studies using the same stimuli, implies that the sample has a role to play; this could be investigated in future research by measuring IQ and assessing its interaction with context. In addition, the stimuli here were static and lacked the complexity of real‐world encounters, where emotions are dynamic and often fleeting. Research indicates autistic people show different patterns of brain activation when viewing static and dynamic stimuli (Pelphrey et al., 2007), and are more likely to attend the faces of static rather than dynamic stimuli (Speer et al., 2007). This suggests that complexity influences how stimuli are attended and processed.

The effect of context may also have differed depending upon the emotion being depicted. Due to the nature of the task used in the present study (one display of each emotion per level of context), further analysis of specific emotions would be underpowered. It must be acknowledged, however, that the emotions presented can influence emotion recognition accuracy, as seen in previous work which shows some difficulties may be emotion specific (Black et al., 2017; Harms et al., 2010; Sowden et al., 2021).

Additional research into emotion recognition and context is needed, which addresses both issues. This has begun in studies with children with autism and the non‐autistic adult population (see Martin et al., 2019; Metcalfe et al., 2019). These studies use CGI animated videos and reveal that an autism diagnosis, increased autistic‐like‐traits, and a lack of contextual cues lead to less accurate emotion perception. These studies however, are devoid of facial expression and rely solely on body movement and gesture; both facial and body expressions should be integrated in future research, to create a more ecologically valid investigation of emotion. In addition, understanding the role of context at different stages of the developmental trajectory of emotion recognition, in autistic people, may prove valuable. This may extend the understanding the socio‐emotional development of autistic people, while also informing the development of effective interventions with long‐lasting and generalisable effects (Kouo & Egel, 2016).

### Limitations

The first limitation relates to the generalisability of the results. As only a subset of autistic people was included, it cannot be assumed that these results apply to all autistic people. A second limitation was that the participants' autism diagnoses were based on self‐report. While many autistic participants were recruited through a charity, that required evidence of diagnosis to provide support, the diagnostic status of those who were recruited via social media is less certain. The AQ scores of the latter group, in‐part supported this self‐report, but several participants did not complete the AQ. A third limitation was that the study used static emotion stimuli and the use of hand‐drawn cartoon‐like imagery in the no‐context condition, rather than ecologically valid and dynamic stimuli. As shown by Rosset et al. (2008) autistic people often find cartoon‐like expressions easier to decode than more realistic expressions, however, no evidence to that effect was found here. While results here may not extrapolate to encounters of emotion displays in the real world, these stimuli were chosen to reflect materials commonly used in assessments of, and interventions, for emotion recognition (e.g., Wood & Kroese, 2007). It is important to explore the impact of context in these types of stimuli to inform future interventions. A further notable point is the group sample size imbalance, specifically that the group of autistic people was smaller than the non‐autistic group. While this can poses an issue for some analysis strategies, it is not necessarily a cause for concern when multilevel modelling is applied (see Twisk, 2006); this is the principal reason for its application in the present study.

## CONCLUSION

These results indicate that autistic adults are less accurate on tasks of emotion recognition than non‐autistic adults. Gender and contextual information were not found to influence emotion recognition accuracy. While this study adds to the limited research on the topic of autism, emotion recognition and context, further research is required. This would aim to ascertain whether context impacts specific emotions, as well as implementing more ecologically valid, dynamic stimuli.

## Data Availability

Data and supplementary materials available on the Open Science Framework (OSF) at https://osf.io/gm9eu/.
